# Memristor Synapse—A Device-Level Critical Review

**DOI:** 10.3390/nano16030179

**Published:** 2026-01-28

**Authors:** Sridhar Chandrasekaran, Yao-Feng Chang, Firman Mangasa Simanjuntak

**Affiliations:** 1 Micro and Nano Devices Laboratory, School of Electronics Engineering, Vellore Institute of Technology, Chennai 600127, India; sridhar.c@vit.ac.in; 2 Microelectronics Research Center, The University of Texas at Austin, Austin, TX 78758, USA; 3 School of Electronics & Computer Science, University of Southampton, Southampton SO17 1BJ, UK; 4 Centre for Neuromorphic Technologies, University of Southampton, Southampton SO17 1BJ, UK

**Keywords:** memristor synapse, STP, LTP, SRDP, STDP

## Abstract

The memristor has long been known as a nonvolatile memory technology alternative and has recently been explored for neuromorphic computing, owing to its capability to mimic the synaptic plasticity of the human brain. The architecture of a memristor synapse device allows ultra-high-density integration by internetworking with crossbar arrays, which benefits large-scale training and learning using advanced machine-learning algorithms. In this review, we present a statistical analysis of neuromorphic computing device publications from 2018 to 2025, focusing on various memristive systems. Furthermore, we provide a device-level perspective on biomimetic properties in hardware neural networks such as short-term plasticity (STP), long-term plasticity (LTP), spike timing-dependent plasticity (STDP), and spike rate-dependent plasticity (SRDP). Herein, we highlight the utilization of optoelectronic synapses based on 2D materials driven by a sequence of optical stimuli to mimic the plasticity of the human brain, further broadening the scope of memristor controllability by optical stimulation. We also highlight practical applications ranging from MNIST dataset recognition to hardware-based pattern recognition and explore future directions for memristor synapses in healthcare, including artificial cognitive retinal implants, vital organ interfaces, artificial vision systems, and physiological signal anomaly detection.

## 1. Introduction

Currently, the world is becoming increasingly dependent on artificial intelligence (AI) computations. AI-powered chips are not only used for supercomputers and cloud servers but also for personal gadgets and computers, which modern life and the global economy cannot run without. The development of these chips mainly focuses on advanced architectures, e.g., graphic and tensor processing units and AI-specific accelerators, and they stimulate the progress of deep learning due to their high computing speed [1]. Nevertheless, the current computer architecture is based on conventional von Neumann designs that rely on data transfer between the processing unit and memory unit, and its transfer rate is facing a limit as the task complexity increases [2]. This von Neumann bottleneck limits the processing speed due to the shared system bus between the CPU and memory, restricting simultaneous instruction fetching and data processing [3]. This problem makes the AI chips consume high energy and limits their efficiency and scalability [1].

Computing-in-memory (neuromorphic computing) is a new paradigm to solve this problem, where the data do not necessarily have to be processed at the processing unit but at the memory unit itself, mimicking how the human brain works. The brain is the most efficient computing system capable of performing complex data processing, enabled by a large-scale interconnected neural network that stores and processes data at the same place and in parallel [4]. This interconnected neural network is governed by synapses to perform synaptic functions [5].

**Figure 1:**
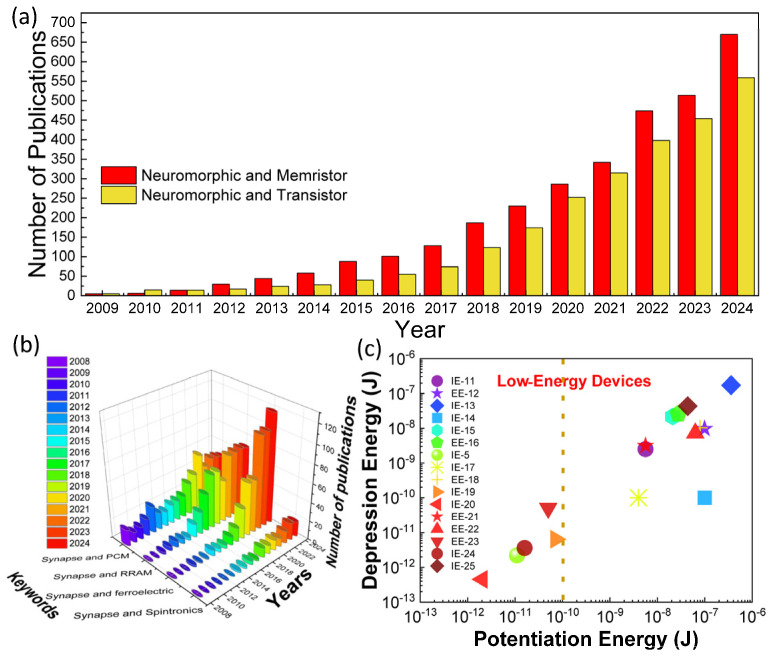
(**a**) Number of publications in neuromorphic computing based on memristor and transistor technologies; (**b**) number of publications of artificial synapses based on various memristive systems; (**c**) memristor synapse exhibiting lower energy operation using insulator engineering (IE) and electrode engineering (EE) [6,15,16,17,18,19,20,21,22,23,24,25,26,27,28,29]. Data in (**a**,**b**) were obtained from webofscience.com.

Several memristive systems, memristor, phase-change, spintronic, and ferroelectric memory devices can mimic the synaptic functions, showing potential for neuromorphic computing architectures; thus, they are called artificial synapses [6,7,8,9,10,11]. Research on neuromorphic computing based on memristive systems has exponentially increased over the last decade, as shown in Figure 1. This trend indicates the urgent demand for faster and smarter computing machines in the real world. Among these memristive systems, it has been shown that phase-change and resistive memories are the most prominent subfields showing rapid growth in development (Figure 1b). However, phase-change memory technology has a long history of scientific contributions, and its development is much more mature [12]. Ferroelectric and spintronic memory devices have been developed over the last few decades [12]. The scientific progress in these fields is not as fast as that in resistive memory. One of the reasons why resistive memory has gained considerable attention is its feasibility for integration with a diode selector, which has the same sandwich structure as a memristor (1D1M, 1-diode 1-memory). In circuit-level implementation, a selected device must be stacked with each memristive system to avoid the sneak-path issue in a massive array configuration. All memristive systems except memristors require transistors as their selectors (1T1M, 1-transistor 1-memory) [13]. The transistor cell structure (10–30F^2^, where F is the feature size) is the constraining factor hindering the fabrication of an ultra-high-density synaptic network, whereas the 1D1M configuration has a cell size of 4F^2^/N (N is the number of stacked layers), which is promising for a large artificial neural network (ANN) [14]. The facile architecture of the two-terminal memristor synapse offers a brain-inspired, highly dense computing element that complies with Moore’s law [2,6,13,15,16,17,18,19,20,21,22].

An ANN employing a 1024-cell array of memristor synapses (memristor-based synaptic devices) consumes much less power than the Intel Xeon Phi processor [6]; von Neumann’s consumes more energy for cognitive learning and data processing, not only because of the CPU memory bottleneck but also because its transistor-based processing unit is volatile and requires continuous power to maintain its synaptic weight [30]. Moreover, ANNs should be integrated with CMOS-based neuron-driving circuits, which are power-hungry [31]. A feasible method to achieve low-power computation is to utilize a low-power ANN based on memristor synapses. The synaptic weight of a memristor synapse can be maintained over a period without continuous power (nonvolatile), and its programming voltage and current are typically 1 V and 10 µA or less, respectively. If typical memristors require 1–3 nJ per spike [32] and 10^8^ spikes are needed for the full 10,000-image MNIST test set (considering about 10^3^ spikes per neuron across 10^4^ neurons firing during training and inference cycles) [33], we can hypothetically estimate that the memristors only consume less than 1 J when scaled to large arrays of memristor devices. On the other hand, if the Xeon Phi processor runs digital CNN-based MNIST and assuming it consumes 225 W under full load, it requires 25 kJ just for one training session [34]. Therefore, the memristors can reduce the overall system-level power consumption by 4 to 5 orders of magnitude [2,35]. Furthermore, the operating power of the memristor synapse can be further reduced by reducing the cell size [36], stacking in a 3D configuration [37,38,39], and insulator and electrode engineering [40,41]. Based on the trend of the energy required to produce synaptic plasticity, shown in Figure 1c, we found that insulator engineering is an effective approach to reduce the power consumption. Nevertheless, memristor synapses have several challenges that hinder their large-scale integration, e.g., device variability, nonlinear and asymmetric weight update, and epoch training variability. This review article discusses these challenges and overviews recent approaches to mitigating them.

## 2. Architectonics and Device Physics

**Figure 2:**
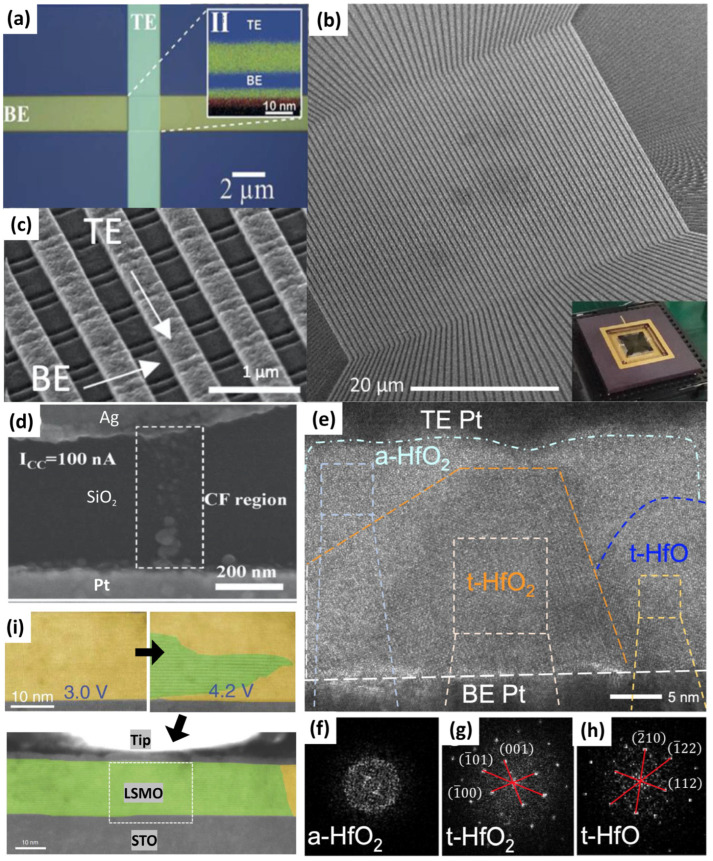
(**a**) Typical architecture of two-terminal metal/insulator/metal memristor synapse, adapted from [84] and (**b**,**c**) high-density 64 × 64 memristor crossbar array configuration with a total of 4096 memory cells, adapted from [16], (**d**) cation-based filamentary formations, adapted from [65], (**e**–**h**) anion-based filamentary formations observed using TEM, adapted from [73], (**i**) homogeneous switching was observed using in situ TEM, adapted from [78].

The basic architecture of a memristor synapse is a two-terminal metal (top electrode)/insulator (switching layer)/metal (bottom electrode) sandwich structure, as shown in Figure 2a. Among various two-terminal configurations, such as planar (both electrodes face each other with a nanometer gap separation) [42], points (the electrodes have crossed [42], circular [28], or rectangular shapes [43], where each cell does not share its electrode(s) with other cells), and crossbars [16]. The crossbar configuration is the most feasible way to achieve a massive ANN because the cells share both the bottom and top electrodes along the intersecting line array, facilitating interconnection between cells (Figure 2b,c).

The synaptic weight of a memristor synapse is controlled by the electrochemistry of the ionic species in the insulator, which drift under the influence of an electric field to build a conduction channel in the switching layer [44]. These ionic species can be cations (metal ions: Ag [45,46,47], Cu [48,49,50], Ni [51], Co [52], In [53,54,55,56], Te [57], or Pd [58]), anions (non-metal ions, oxygen [59], nitrogen [60,61], carbon [62], and their vacancies), or a combination of both (hybrids) [63,64]. Cation-based memristor synapses, also known as conducting-bridge memory or electrochemical metallization memory (ECM), rely on the migration of metallic ions originating from the active electrode (as the cation source) into the switching layer, forming a bridge connecting the bottom and top electrodes. Figure 2d depicts the direct growth of Ag nanobridges in the Ag/SiO_2_/Pt ECM cell [65]. Nevertheless, in some cases, an inert metal may also migrate and form a bridge [66], which could be due to the property of the film affecting the mass transport under an electric field [67]. The geometry of the metallic bridge is governed by film inhomogeneity and electric field distribution, which determine the redox rate, nucleation rate, and ionic mobility of the metal ions. For example, high rates and mobility result in an inverted cone-shaped filament that grows from the inert electrode, whereas low rates and mobility result in discrete nanoclusters growing from the active electrode, forming a forward cone shape [68]. However, if no active electrode is used in the device, the percolation of intrinsic donor defects in the switching film may form a conducting filament because of the drift of anions under an electric field. In metal oxide (MO) and metal nitride (MN) systems, the filament consists of oxygen (V_O_^2+^) and nitride (V_N_^+^) vacancies. The filament is formed by the reduction of oxygen or nitrogen atoms, forming O^2−^ or N^3−^ anions, respectively, where these anions move to the anode and create vacancies (V_O_^2+^ or V_N_^3+^) in the system [69,70]. Other possible ionic vacancy configurations are also possible, depending on the insulator and electrode materials [71,72]. Thereafter, these vacancies are ordered to connect both electrodes as a pathway for electrons to flow from the cathode to the anode.

Several studies have suggested that the arrangement of these vacancies in crystal lattices may induce a phase transformation, forming a highly conducting phase in the switching layer, such as the Zn-dominated ZnO_1−x_ and Ti_4_O_7_ Magnéli phases in ZnO and TiOx systems, respectively; in some cases, it can also induce amorphous-to-crystalline transition to form a conducting filament(s), as depicted in Figure 2e–h [73]. Another piece of compelling evidence for the phase-change phenomenon occurred in the SiO_x_ system, where the filament is in the form of a semi-metallic Si state [74]. A similar phenomenon was also observed in the SrTiO_3_ system; it was found that the formation of Sr-rich secondary phases led to a high current density [59]. It is important to note that no Sr or Ti ionic movement is involved in the switching process. In hybrid-based devices, both anion and cation species are involved in the switching operation, where the filament and bridge coexist to build a complete conduction channel connecting the electrodes [50,75]. Metal cations have a smaller radius and higher mobility than anions; thus, cations may reach the cathode faster, and the bridge structure has a larger portion of the channel than the filament [64]. Nevertheless, the involvement of various species in a complex system, such as memristor synapses with an active metal-doped oxynitride system, requires further investigation to explain the contribution of each defect to channel formation and its impact on synaptic stability.

In contrast to filamentary conduction, an electrochemical reaction can occur in most parts of the bulk switching layer, which is known as a homogeneous or interfacial switching mechanism. This non-filamentary conduction is similar to the anion-based mechanism; in the ZnO-based system, for example, the repulsion of anions (O^2−^) occurs in a much larger region in the bulk, where this process creates a conducting (oxygen-poor) region above the anode and an insulating (oxygen-rich) region below the cathode in the switching layer [76]. In the ABO_3−x_ system, the electrochemical reaction induces a brownmillerite-perovskite phase transformation, a phenomenon known as reversible phase change [77]. Figure 2i depicts the phase transformation process in the LaSrMnO system [78]. Contrary to most metal-oxide systems, the conducting (perovskite) and insulating (brownmillerite) regions in ABO_3−x_ are oxygen-rich and oxygen-poor phases, respectively [79]. Electron transport in LaSrMnO relies on the hopping of electrons via Mn^4+^ and Mn^3+^ cations, and the introduction of oxygen shifts the under-doped phase (insulating) towards the effective doping level, thereby decreasing the electrical resistance [78].

Based on the above discussion, the anion-based filamentary and homogeneous switching behaviors rely on electrochemically induced phase transition. Therefore, we can argue that the physical nature of the filamentary and interfacial mechanism is not simply a pile of oxygen vacancy defects providing electron pathways between the two electrodes; rather, they rely on the transformation of an insulating phase into a conducting phase that occurs in the switching layer.

A similar phenomenon is observed in phase-change memory technologies; however, the difference is that the switching mechanism in phase-change memory relies on a heat-induced phase transition from crystalline to amorphous, which means this process breaks the lattice frames and vice versa [80]. Nevertheless, this similarity brings us to question the fundamental terminology: can we classify anion-based filamentary and homogeneous memristor synapses as a class of (localized) phase-change memory?

A three-terminal structure, known as a memtransistor, was also proposed for fabricating memristor synapses [81]. Unlike the two-terminal memristor synapse, the gate in the memtransistor offers additional control to modify the conduction between the terminals [82]. Although the memtransistor offers operational ability to minimize the sneakpath current across a large-scale array and mitigate device variability [83], the three-terminal device occupies a larger area than the two-terminal crossbar. Thus, this architecture limits high-density 3D integration. Nevertheless, the fabrication flow complexity of the memtransistor architecture is much less than that of any two-terminal memristive synapse with a transistor as the selection device (1T1M).

## 3. Synaptic and Neuromorphic Capabilities

**Figure 3:**
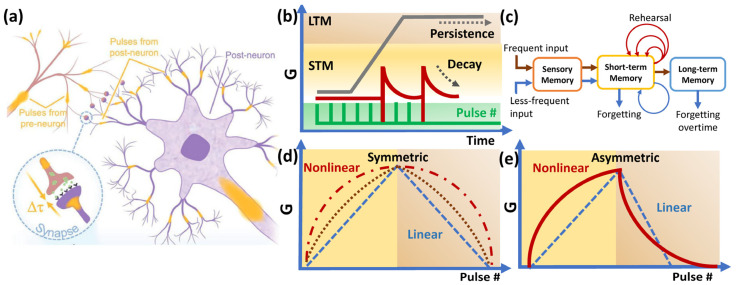
(**a**) Schematic of synapses, reprinted from [92]; (**b**,**c**) short-term and long-term memory effect on memristor synapse with different forgetting behavior, inspired by [89]; (**d**,**e**) symmetric and asymmetric synaptic feedback exhibiting linear and nonlinear actuation.

The mammalian brain consists of interconnected neurons that can process complex data via an electrochemical firing mechanism at synapses [85]. Synapses are junctions that connect each neuron in the network, as depicted in Figure 3a, and transfer and receive chemical signals (Ca^2+^, Na^+^, or K^+^ ions, known as neurotransmitters) [86]. Neurotransmission is mediated by the release of glutamate from a presynaptic neuron (the neuron or synapse that sends the neurotransmitter) to a postsynaptic neuron (the neuron that receives the neurotransmitter). Postsynaptic neurons contain AMPA receptors, which influence Ca^2+^ influx, leading to synaptic plasticity [87,88]. Depending on how the presynaptic fires the transmitter and the postsynaptic response towards the incoming signal, the network can exhibit long-term and short-term memorization, which we define as neural plasticity [89,90].

Similarly, the firing mechanism of memristor synapses was achieved using electrical pulses, and the synaptic response was measured in terms of conductivity, as shown in Figure 3b. In this case, the cathode acts as the pre-synapse and the anode as the post-synapse, whereas the mobile cation/anion in the insulator layer acts as the transmitter (Figure 2). Various pulse schemes can be used to program memorization (synaptic strength); typically, a slow rate and low-amplitude stimulus will result in short-term memory (STM) that can retain the response for a short period (sub-milliseconds to minutes), after which the device starts to forget, followed by a rapid decay of conductance, as illustrated in Figure 3c [91].

**Figure 4:**
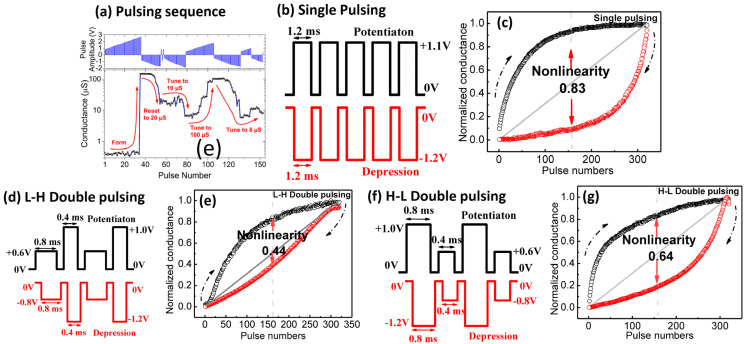
(**a**) The typical pulsing sequence of the pristine memristor synapse, reprinted from [16]; the pulsing sequence on applying LTP and LTD of (**b**,**c**) identical pulses (single pulsing), (**d**,**e**) low–high double pulsing, and (**f**,**g**) high–low double pulsing schemes, reprinted from [17].

STM can evolve into long-term memory (LTM, hours to days) upon high-frequency stimuli or vice versa via opposite amplitudes. The rehearsal and forgetting mechanisms are similar to how the brain learns and processes information [87,88]. The decay rate of synaptic weight can be natural (intrinsic decay), programmed, or a combination of both. The increase and decrease in synaptic weight are called potentiation and depression processes, respectively, and the a/symmetricity of the potentiation and depression can be a linear or nonlinear response, as depicted in Figure 3d,e. Ionic or organic-based memristor synapses often exhibit symmetric feedback, but these types of devices are less stable for lifelong deployment; meanwhile, metal oxide-based devices are more durable but have asymmetric feedback because of the physical nature of the growth and dissolution of the conduction channel in the oxide [93]. Symmetrical synaptic feedback offers a more straightforward computing algorithm at the system level, and it is suggested that linearity determines the computing accuracy [94]. Most metal-oxide memristor synapses require soft breakdown (electroforming) to activate synaptic feedback (Figure 4a) [16]. Different pulse schemes produce different synaptic feedback; Figure 4b–f show the typical identical pulse (pairs) and nonidentical pulse schemes, and the nonidentical pulse often offers better linearity than its identical counterpart [2,16,17]. Linearity can also be enhanced by material and interface engineering [2,16]; it is suggested that a multilayer switching film is an effective method to improve the linearity, since each layer has unique nanostructure and defect properties; thus, controlling the filament or bridge configuration during the synaptic process becomes easier [95,96]. However, there is still less research on the impact of nonlinear and asymmetric feedback on the speed and accuracy of ANN computations in actual implementation.

Besides nonlinearity, temporal variation, such as conductance drift and the dynamic range disparity, which often occur due to cycle-to-cycle and device-to-device fluctuation, can significantly affect the learning accuracy of the network. It is suggested that a network with a temporal variation of more than 22% would not be able to achieve 90% learning accuracy [97]. Device stacking techniques have been proposed to mitigate the temporal variation, e.g., 2M1T (a series of two memristor synapses connected with a transistor) [98], or place several memristors in parallel to suppress the random rejuvenation of the filament during the synaptic training process [99].

**Figure 5:**
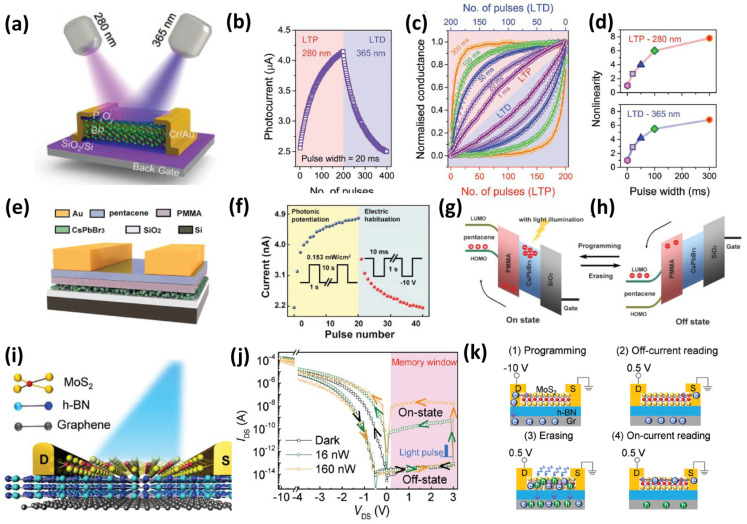
Optoelectronic synapse UV light-based switching (**a**) schematic of BP memtransistor and its (**b**) optical LTP and LTD, (**c**) weight change on varying the light pulse width, and (**d**) the nonlinearity of the LTP and LTD at different wavelengths and pulse widths, adapted from [100]; (**e**–**h**) optically activated CsPbBr_3_ memtransistor showcasing the potentiation and depression with ON state and OFF state band alignment, adapted from [101]; (**i**–**k**) optical response of two-terminal MoS_2_ memristor synapse and its mechanisms; a higher light power increases the number of generated electrons, adapted from [102].

Optical illumination can also induce or facilitate synaptic responses. Ref. [100] reported the optical stimulation of a BP-based memtransistor using 280 nm and 365 nm UV light for potentiation and depression, respectively, as shown in Figure 5a,b. The pulse width of the light stimuli can be adjusted to tune the linearity (Figure 5c,d). Yan et al. [101] suggested that light stimuli trigger charge trapping and release mechanisms in the CsPbBr_2_ synapse (Figure 5e–h). A similar mechanism can be found in the two-terminal planar Graphene/MoS_2_ memristor synapse, where the light pulses induce photogenerated trapped electrons, thus enabling programming and erasing functions in the device (Figure 5i–k) [102]. Metal oxide-based memristor synapses with point configurations can also produce photo-induced synaptic behavior, suggesting that the mechanism behind the increase or decrease in synaptic weight during light stimulation is due to the ionization and neutralization of oxygen vacancies in the oxide layer, respectively [103]. These devices require transparent electrodes, such as indium tin oxide (ITO), or a thin metal layer to enable light to penetrate the electrode into the oxide layer. It was found that various wavelengths, from ultraviolet to visible to infrared, can induce a photosynaptic response. Note that ultraviolet light is most commonly used to trigger the photosynaptic response owing to its high energy. Nevertheless, further investigations should be conducted to clarify or confirm the mechanism behind the photosynaptic response in devices with ITO electrodes. The high absorption of ITO in the UV region absorbs light before it can be transmitted into the oxides; thus, this brings us another idea that we may not necessarily expose the insulator layer if we can employ photosensitive electrode materials.

Table 1 summarizes the performance metric of optomemristor and optomemtransistor synapses. Both the optomemristor and optomemtransistor are suitable for visual AI systems due to their acceptable optical speed. Even though optomemtransistor synapses could perform with better accuracy in conductance modulation and fast photoresponse and high responsivity, their engineering complexity and integration may result in a higher fabrication cost than that of optomemristors. The use of perovskite or 2-dimensional materials tends to lead to a better optical performance [104]. However, these materials are often sensitive to ambient aspects, e.g., temperature and humidity, that can pose challenges for practical use [105,106,107]. Table 2 summarizes common problems in developing 2-dimensional memristor synapses. A careful and tedious synthesis route is required to grow these materials, and slight process variation in the nanostructure or defect configuration could significantly affect their characteristics and performance.

**Figure 6:**
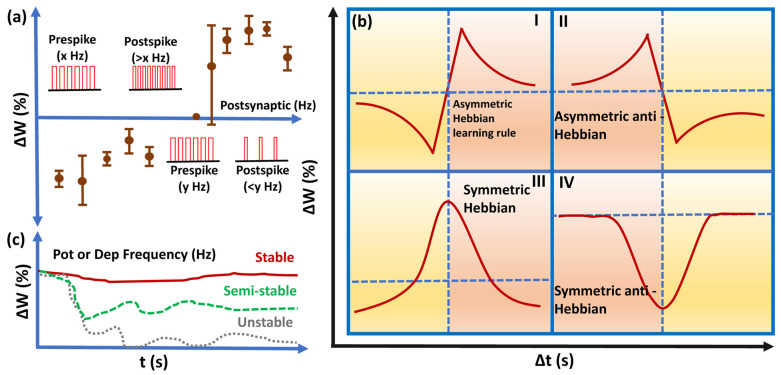
(**a**) Hebbian learning rules for the symmetric and asymmetric responses, inspired by [127]; (**b**) schematic of SRDP programming procedure employing various frequencies for the pre- and post-spike response and (**c**) spike frequency of the SRDP algorithm determines the synaptic weight, inspired by [43].

Remembering and forgetting are neuronal functions that neuromorphic computing systems try to mimic [121] and are deeply rooted in the learning rules that are used to modulate synaptic weights. A neurophysiologist, Donald Hebb, proposed that synaptic weight changes in biological neural networks may vary depending on the time gap between spikes, known as Hebbian learning rules (HLRs) [122], the most common of which are shown in Figure 6a. Spike timing-dependent plasticity (STDP) and spike rate-dependent plasticity (SRDP) are frequently used as programming algorithms for implementing these rules [123]. STDP works on the relative timing between the pre- and postsynaptic spikes and influences the magnitude and direction of the weight update [124], whereas SRDP works on synaptic weight modulation by controlling the frequency of spiking neurons in the neural network, as illustrated in Figure 6b. Although STDP is mostly reported in memristor synapse development owing to its practical implementation in decreasing or increasing the weight with precise timing of each spike, SRDP is closer to biological neuronal functions, where the spike rates control synaptic strength (Figure 6c). Peng et al. [125] suggested that the SRDP is an efficient algorithm for high-accuracy unsupervised online learning. Ideally, a memristor synapse should be able to perform HLRs. Yi Li et al. [126] reported that a Ge_2_Sb_2_Te_5_-based memristor synapse can exhibit all four rules with fast response time and low-power operation. However, most memristor synapse designs in the literature fail to demonstrate HRLs.

## 4. Hardware Implementation and Future Scope

Recent advancements in memristor-based neuromorphic systems have enabled a wide spectrum of biomedical applications. These can be broadly categorized based on the nature of the signal they process—optical signals for vision restoration, electrochemical signals for organ-level implants, and electrophysiological signals for wearable health monitoring. Additionally, these applications vary in their level of invasiveness, ranging from fully implantable devices to non-invasive wearable electronics. This section explores these applications in a structured manner to highlight the synergy between device-level innovations and system-level healthcare solutions.

### 4.1. Optical Signal Processing: Artificial Retinas and Vision Systems

**Figure 7:**
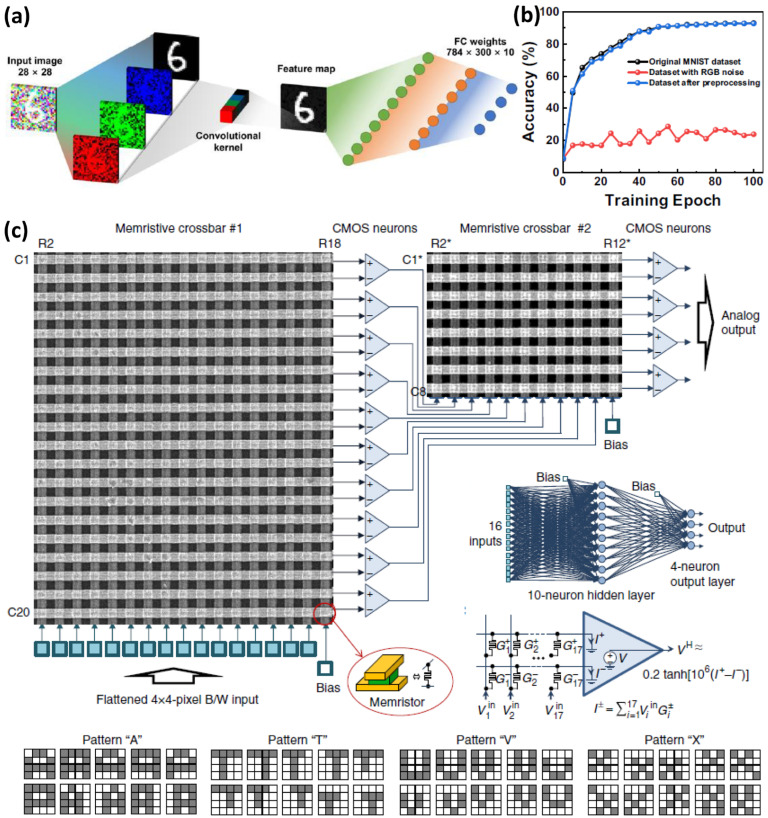
(**a**) Neural network simulation of the MINST dataset handwritten numbers with and without RGB noise; (**b**) recognition accuracy simulation of the neural network, adapted from [128]; and (**c**) high-density crossbar memory array for pattern recognition of various character patterns, adapted from [132].

Li et al. [128] proposed a VO_2_-based ultraviolet nonvolatile synapse memtransistor actuated by 650, 532, 450, and 375 nm light sources. The synapse response of the memtransistor was used for neural network simulation to recognize handwritten numbers from the MNIST dataset. Figure 7a depicts the single-layer feedforward artificial neural network with 784 neuron input layers, 300 neuron hidden layers, and 10 neuron output layers presented in this network. The neural network was trained using the original MNIST test dataset, the RGB Gaussian noise dataset, and preprocessed data. The neural network recognition accuracy was simulated as shown in Figure 7b. The preprocessed dataset and the original MNIST test dataset achieved a recognition accuracy of 93%, whereas the dataset with RGB Gaussian noise achieved only 24% accuracy. The hardware implementation of a multilayer perceptron (MLP) neural network was realized by Bayat et al. by developing a 20 × 20 memristor synapse crossbar array based on Pt/Al_2_O_3_/TiO_2−x_/Ti/Pt. The MLP consists of 16 input neurons, 10 hidden layer neurons, and 4 output neurons. The hardware-based neuron network was trained to classify 4 × 4 pixel patterns, shown in Figure 7c. MNIST simulations based on memristor synapses typically achieve 90–95% accuracy; this performance is close to the accuracy achieved by traditional ANN software-based or other hardware, which could achieve up to 99% [129,130,131].

**Figure 8:**
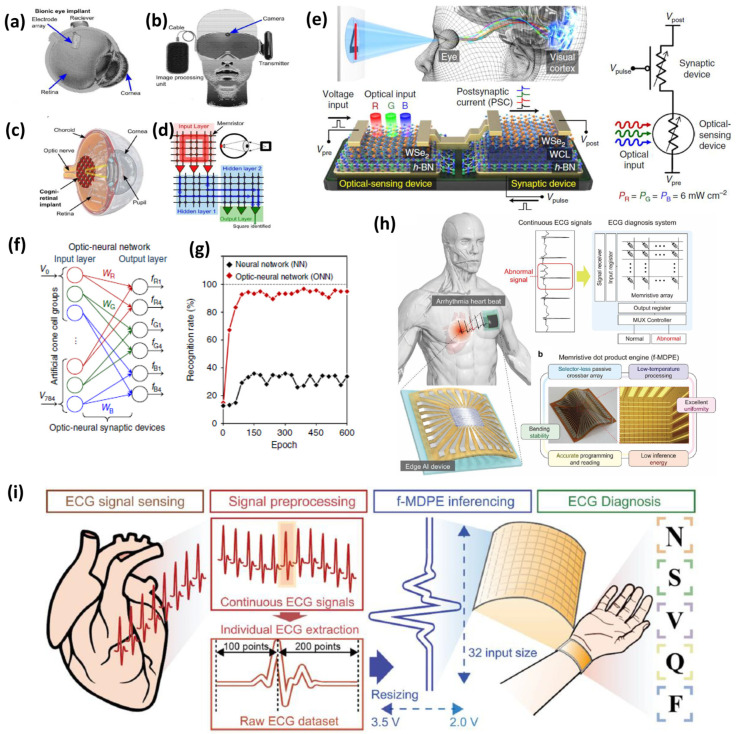
(**a**–**d**) The concept of bionic retinal implant employing HNN, adapted from [135]; (**e**) integrated optical sensing and synaptic devices mimicking human optical nerve system and (**f**,**g**) RGB-based pattern recognition on optic-neural network, adapted from [136]; (**h**) human body-based various implantable electronics based on HNN; and (**i**) vital parameter signal monitoring and diagnosis using memristor, adapted from [137].

In recent years, implantable electronics has grown rapidly owing to the significant leaps achieved by various artificial vision projects worldwide [133,134]. An artificial cognitive retina is based on a biocompatible synaptic array, such as a retinal implant, to transmit images efficiently into an optical neuronal signal. Retinal implants are directly connected to the optic nerve in the human brain. The main function of optical nerves in humans is to transmit visual data obtained from the rod and cone cells of the retina to neuronal signals of the human brain, which are then processed by the human brain as visual signals. Figure 8a depicts the location of the retinal implant connecting the optical nerves and retina [135]. The image from the camera was processed using a 3D stacked memristor array, which was then transferred to the retinal implant, as shown in Figure 8b,c. The 3D stacked crossbar memristor that processes the image data into optical nerves as a visual impulse. The artificial neural network comprises two hidden-layer biocompatible synaptic memristor arrays for converting electronic data (image data) to chemical translation (optical nerves). The structure of the neural network is shown in Figure 8d. The first layer of this neural network array was employed to capture a visual image and imprint it into an array of artificial neural networks.

The successive layer participates in the computations to further conduct the image processing operation for conversion into an optical signal. The primary function of the optical nerves is to convert images into signals. Retinal implants are expected to help improve the lives of patients with visual disabilities. Seo et al. proposed an artificial optic-neural synapse [136] that contributes to a significant enhancement in synthetic vision by directly interfacing an optical sensing device with a synaptic device connected to the visual cortex of the human brain. The structure of the integrated h-BN/WSe_2_ (optical sensing) and WSe_2_-based (synaptic device) is shown in Figure 8e. The schematic also illustrates a symbolic representation of the connected device used in the study. The synaptic device is expected to be integrated into an array of neural networks for high-density integration and realization of artificial vision. The synaptic device response can be used in an optical neural network of RGB-based synaptic devices, and the neural network can achieve a peak recognition rate that exceeds 90% Figure 8f,g. While optical signal processing is crucial for restoring vision, similar neuromorphic architectures can be adapted for electrochemical interfaces in vital organ implants. These systems enable real-time monitoring and data interpretation directly within the body.

### 4.2. Electrochemical Interfaces: Organ-Level Implants

In the future, memristive HNN cannot be restricted to bionic vision in the healthcare sector. HNN can be utilized in various applications, including heart, brain, ovarian, and lung implants, which are integrated with HNN, as depicted in Figure 8h,i. Artificial neural networks could dominate the future of biomedical applications, from heart implants to human brain implants. The future of biomedical analysis can be carried out using heart or brain implants with sensors and an HNN to harness the data processing of the functionality of human organs. The HNN can also categorize patients’ diseases using the data available from implants. Memristor synapses leveraging electrochemical interfaces are groundbreaking for organ-level implants, as they mimic biological synaptic function through ionic and redox-driven resistance switching, thus enabling ultra-low-power and scalable architectures for physiological data processing [138]. Hardware neural networks incorporating memristor crossbar arrays can process multichannel physiological signals from these implants by applying learning algorithms like spike-timing and rate-dependent plasticity, supporting responsive closed-loop interventions directly at the organ interface. Implanting memristor synapses on the heart could facilitate real-time, energy-efficient detection of irregular cardiac activity by encoding and evaluating biosignals locally [139]. For brain implants, memristor synapses allow high-fidelity neural recording and the adaptive neuromodulation necessary for cognitive rehabilitation, via robust hardware neural network architectures; similarly, ovarian implants can exploit memristor synapses for continuous hormonal cycle monitoring, supporting both diagnosis and therapy through on-site computation with minimal energy overhead [140]. Another successful proof-of-hypothesis has also been reported for applications in functional monitoring of blood supply during liver transplantation [141]. This enables implantable electronics to perform real-time classification, anomaly detection, and feedback using integrated neuromorphic architectures proven to work in biomedical scenarios [142]. Beyond invasive implants, neuromorphic computing also supports non-invasive health monitoring through wearable electronics. These devices serve as external hubs, collecting and analyzing electrophysiological signals to provide continuous health insights.

### 4.3. Electrophysiological Monitoring: Wearable Sensors

Memristor synapses are revolutionizing non-invasive health monitoring through their potential integration into wearable electronics for ECG, SpO_2_, heart rate, and blood pressure measurement [137]. Memristors can act as external hubs, continuously collecting electrophysiological signals and leveraging neuromorphic architectures for low-latency data processing [143]. The use of memristor-based in-memory computing within these wearables allows real-time analysis and pattern recognition directly at the sensor level, which is essential for anomaly detection such as arrhythmias or abnormal blood oxygenation [144]. By embedding computational functions into the storage medium, memristor synapses minimize energy consumption, enable scalable data fusion, and facilitate instant feedback to healthcare providers or end-users, ensuring timely intervention and improved patient outcomes [145]. Wearable electronics, which could act as a data hub to connect these implants, demonstrate the live functionality of the human organs and display the data using transparent electronic eyeglasses or notify doctors. By using a similar approach, Ni et al. [146] proposed FeFET-based sensor data fusion technology using in-memory or neuromorphic memory computing. The FeFET-based neural network was designed to detect anomalies in the live ECG, SpO_2_, heart rate, and blood pressure of human physiological signals. An in-memory logical array was designed to monitor and analyze human physiological signals. The in-memory correlation detection array is composed of multiple sensors that feed input to the correlation detection system, which is composed of a neural network of processing in the FeFET, and the anomaly detection scheme is used to detect anomalies in physiological signals.

## 5. Conclusions

Resistive-synapse-based neuromorphic computing offers great potential for the development of next-generation AI hardware. The mimicking property of memristor synapses allows for the emulation of cognitive activity similar to that of the human brain, which exhibits STDP and SRDP. Research on highly dense crossbar array-based HNNs shows a significant leap by realizing a simple perceptron neural network proposed to perform various applications based on recognition, classification, and detection. In the future, we anticipate that HNN-based systems will be used for broad healthcare solutions, including artificial vision, EEG signal monitoring, vital organ implants, disease detection, and physiological signal monitoring. Nevertheless, system-oriented benchmarking for memristor synapses should be developed to determine the minimum required synaptic performance for certain applications to ensure better synergy and a scientific leap between cleanroom engineers (device level) and system designers (system level). We also foresee further scientific progress in memristor synapses that mimic various types of neurons by controlling defects to behave like biological neurotransmitters. Therefore, the development of the next generation of memristor synapses will extend beyond elemental-to-molecular-level neural networks with massive interconnections close to the human brain.

## Figures and Tables

**Table 1:** Typical performance metrics of optomemristor and optomemtransistor synapses in the published literature.

Performance Metrics	Optomemristor	Optomemtransistor
Energy per optical spike	Sub-fJ to few fJ [108]	As low as 0.03 fJ per operation [109]
Programming speed (spike width, photocurrent decay)	Slow, seconds [110]	Fast, milliseconds [111]
Spectral selectivity	Good, can differentiate several bands of wavelength for multi-channel programming [112]	Excellent, can be designed to respond to a narrow band or specific wavelength via bandgap/channel engineering [113]
Optical responsivity	Low, 2.7 A/W [114]	High, 12 A/W [111]

**Table 2:** Typical challenges in the development of 2-dimensional memristor synapses.

Material System	Synaptic Mechanism	Potential Limitations
Pt/MoS_2_/Ti	Thermionic emission	Require uniform wafer-scale synthesis; interface trap states could cause variability; and CMOS integration remains limited [115]
MoTe_2_	Ionic filament and phase transition	Phase instability might occur; sensitive to thermal and moisture; and complex structure limits reproducibility [116]
Al/WS_2_/MoS_2_/ITO	Sulfur ions distribution at the interface	Short retention and endurance; random filament rupture–rejuvenation might limit large-scale integration maturity [117]
Ag/a-BN/Pt	Ag filament and Boron vacancy	Difficult to control the amorphous uniformity [118]
Au/Ti/h-BN/Au	Injection of Ti ions into the system	Switching variability; low endurance, tunneling current may be sensitive to film thickness; and difficult to control the native defects in the atomic layers [119]
Al/Ti_3_C_2_:Ag/Pt	Aggregation of Ag ions around atomic vacancy	Trap density variation can affect linearity [120]

## Data Availability

No new data were created or analyzed in this study.

## References

[1] Hou W. (2024). Exploring the Horizon of AI Development: Navigating Constraints of Chips and Power in the Technological Landscape. Appl. Comput. Eng..

[2] Yu S. (2018). Neuro-Inspired Computing with Emerging Nonvolatile Memory. Proc. IEEE.

[3] Syed G.S., Le Gallo M., Sebastian A. (2024). Non von Neumann Computing Concepts. Phase Change Materials-Based Photonic Computing.

[4] Dittmann R., Strachan J.P. (2019). Redox-Based Memristive Devices for New Computing Paradigm. APL Mater..

[5] Zidan M.A., Strachan J.P., Lu W.D. (2018). The Future of Electronics Based on Memristive Systems. Nat. Electron..

[6] Yao P., Wu H., Gao B., Eryilmaz S.B., Huang X., Zhang W., Zhang Q., Deng N., Shi L., Wong H.-S.P. (2017). Face Classification Using Electronic Synapses. Nat. Commun..

[7] Wu W., Wu H., Gao B., Deng N., Yu S., Qian H. (2017). Improving Analog Switching in HfO_x_-Based Resistive Memory with a Thermal Enhanced Layer. IEEE Electron. Device Lett..

[8] Suri M., Bichler O., Querlioz D., Cueto O., Perniola L., Sousa V., Vuillaume D., Gamrat C., DeSalvo B. Phase Change Memory as Synapse for Ultra-Dense Neuromorphic Systems: Application to Complex Visual Pattern Extraction. Proceedings of the Technical Digest—International Electron Devices Meeting, IEDM 2011.

[9] Han S., Pool J., Tran J., Dally W.J. Learning Both Weights and Connections for Efficient Neural Networks. Proceedings of the Advances in Neural Information Processing Systems 28 (NIPS 2015).

[10] Kaneko Y., Nishitani Y., Ueda M. (2014). Ferroelectric Artificial Synapses for Recognition of a Multishaded Image. IEEE Trans. Electron. Devices.

[11] Burr G.W., Shelby R.M., Sebastian A., Kim S.S., Kim S.S., Sidler S., Virwani K., Ishii M., Narayanan P., Fumarola A. (2017). Neuromorphic Computing Using Non-Volatile Memory. Adv. Phys. X.

[12] Prinzie J., Simanjuntak F.M., Leroux P., Prodromakis T. (2021). Low-Power Electronic Technologies for Harsh Radiation Environments. Nat. Electron..

[13] Salahuddin S., Ni K., Datta S. (2018). The Era of Hyper-Scaling in Electronics. Nat. Electron..

[14] Li C., Belkin D., Li Y., Yan P., Hu M., Ge N., Jiang H., Montgomery E., Lin P., Wang Z. (2018). Efficient and Self-Adaptive in-Situ Learning in Multilayer Memristor Neural Networks. Nat. Commun..

[15] Chandrasekaran S., Simanjuntak F.M., Saminathan R., Panda D., Tseng T.-Y. (2019). Improving Linearity by Introducing Al in HfO 2 as a Memristor Synapse Device. Nanotechnology.

[16] Kim H., Mahmoodi M.R., Nili H., Strukov D.B. (2021). 4K-Memristor Analog-Grade Passive Crossbar Circuit. Nat. Commun..

[17] Chandrasekaran S., Simanjuntak F.M., Panda D., Tseng T.-Y. (2019). Enhanced Synaptic Linearity in ZnO-Based Invisible Memristive Synapse by Introducing Double Pulsing Scheme. IEEE Trans. Electron. Devices.

[18] Prezioso M., Merrikh-Bayat F., Hoskins B.D., Adam G.C., Likharev K.K., Strukov D.B. (2015). Training and Operation of an Integrated Neuromorphic Network Based on Metal-Oxide Memristors. Nature.

[19] Serb A., Bill J., Khiat A., Berdan R., Legenstein R., Prodromakis T. (2016). Unsupervised Learning in Probabilistic Neural Networks with Multi-State Metal-Oxide Memristive Synapses. Nat. Commun..

[20] Gupta I., Serb A., Khiat A., Zeitler R., Vassanelli S., Prodromakis T. (2016). Real-Time Encoding and Compression of Neuronal Spikes by Metal-Oxide Memristors. Nat. Commun..

[21] Moon K., Fumarola A., Sidler S., Jang J., Narayanan P., Shelby R.M., Burr G.W., Hwang H. (2018). Bidirectional Non-Filamentary RRAM as an Analog Neuromorphic Synapse, Part I: Al/Mo/Pr_0.7_Ca_0.3_MnO_3_ Material Improvements and Device Measurements. IEEE J. Electron. Devices Soc..

[22] Gao L., Wang I.-T., Chen P.-Y., Vrudhula S., Seo J., Cao Y., Hou T.-H., Yu S. (2015). Fully Parallel Write/Read in Resistive Synaptic Array for Accelerating on-Chip Learning. Nanotechnology.

[23] Cüppers F., Menzel S., Bengel C., Hardtdegen A., Von Witzleben M., Böttger U., Waser R., Hoffmann-Eifert S. (2019). Exploiting the Switching Dynamics of HfO_2_-Based ReRAM Devices for Reliable Analog Memristive Behavior. APL Mater..

[24] Li Y., Fuller E.J., Sugar J.D., Yoo S., Ashby D.S., Bennett C.H., Horton R.D., Bartsch M.S., Marinella M.J., Lu W.D. (2020). Filament-Free Bulk Resistive Memory Enables Deterministic Analogue Switching. Adv. Mater..

[25] Choi Y.J., Kim M.H., Bang S., Kim T.H., Lee D.K., Hong K., Kim C.S., Kim S., Cho S., Park B.G. (2020). Insertion of Ag Layer in TiN/SiN_x_/TiN RRAM and Its Effect on Filament Formation Modeled by Monte Carlo Simulation. IEEE Access.

[26] Wang I., Chang C., Chiu L., Chou T., Hou T.-H. (2016). 3D Ta/TaO_x_/TiO_2_/Ti Synaptic Array and Linearity Tuning of Weight Update for Hardware Neural Network Applications. Nanotechnology.

[27] Dalgaty T., Payvand M., Moro F., Ly D.R.B., Pebay-Peyroula F., Casas J., Indiveri G., Vianello E. (2019). Hybrid Neuromorphic Circuits Exploiting Non-Conventional Properties of RRAM for Massively Parallel Local Plasticity Mechanisms. APL Mater..

[28] Rajasekaran S., Simanjuntak F.M., Chandrasekaran S., Panda D., Saleem A., Tseng T.-Y. (2022). Flexible Ta_2_O_5_/WO_3_ -Based Memristor Synapse for Wearable and Neuromorphic Applications. IEEE Electron. Device Lett..

[29] Jung P.-Y., Panda D., Chandrasekaran S., Rajasekaran S., Tseng T.-Y. (2020). Enhanced Switching Properties in TaO_x_ Memristors Using Diffusion Limiting Layer for Synaptic Learning. IEEE J. Electron. Devices Soc..

[30] Rojas R., Hashagen U. (2002). “Nothing New Since von Neumann”: A Historian Looks at Computer Architecture, 1945–1995. The First Computers: History and Architectures.

[31] Wijesinghe P., Ankit A., Sengupta A., Roy K. (2018). An All-Memristor Deep Spiking Neural Computing System: A Step Toward Realizing the Low-Power Stochastic Brain. IEEE Trans. Emerg. Top. Comput. Intell..

[32] Yuan R., Duan Q., Tiw P.J., Li G., Xiao Z., Jing Z., Yang K., Liu C., Ge C., Huang R. (2022). A Calibratable Sensory Neuron Based on Epitaxial VO2 for Spike-Based Neuromorphic Multisensory System. Nat. Commun..

[33] Ostrau C., Klarhorst C., Thies M., Rückert U. (2022). Benchmarking Neuromorphic Hardware and Its Energy Expenditure. Front. Neurosci..

[34] Viebke A., Pllana S. (2015). The Potential of the Intel (R) Xeon Phi for Supervised Deep Learning. Proceedings of the 2015 IEEE 17th International Conference on High Performance Computing and Communications, 2015 IEEE 7th International Symposium on Cyberspace Safety and Security, and 2015 IEEE 12th International Conference on Embedded Software and Systems.

[35] Lawson G., Sosonkina M., Shen Y. (2015). Towards Modeling Energy Consumption of Xeon Phi. arXiv.

[36] Yang J., Xue X., Xu X., Wang Q., Jiang H., Yu J., Dong D., Zhang F., Lv H., Liu M. (2021). 24.2 A 14nm-FinFET 1Mb Embedded 1T1R RRAM with a 0.022µm 2 Cell Size Using Self-Adaptive Delayed Termination and Multi-Cell Reference. Proceedings of the 2021 IEEE International Solid-State Circuits Conference (ISSCC).

[37] Barraud S., Ezzadeen M., Bosch D., Dubreuil T., Castellani N., Meli V., Hartmann J.M., Mouhdach M., Previtali B., Giraud B. (2020). 3D RRAMs with Gate-All-Around Stacked Nanosheet Transistors for In-Memory-Computing. Proceedings of the 2020 IEEE International Electron Devices Meeting (IEDM).

[38] Li H., Li K.-S., Lin C.-H., Hsu J.-L., Chiu W.-C., Chen M.-C., Wu T.-T., Sohn J., Eryilmaz S.B., Shieh J.-M. (2016). Four-Layer 3D Vertical RRAM Integrated with FinFET as a Versatile Computing Unit for Brain-Inspired Cognitive Information Processing. Proceedings of the 2016 IEEE Symposium on VLSI Technology.

[39] Wang T., Meng J., Chen L., Zhu H., Sun Q., Ding S., Bao W., Zhang D.W. (2021). Flexible 3D Memristor Array for Binary Storage and Multi-states Neuromorphic Computing Applications. InfoMat.

[40] Banerjee W., Liu Q., Hwang H. (2020). Engineering of Defects in Resistive Random Access Memory Devices. J. Appl. Phys..

[41] Zahoor F., Azni Zulkifli T.Z., Khanday F.A. (2020). Resistive Random Access Memory (RRAM): An Overview of Materials, Switching Mechanism, Performance, Multilevel Cell (Mlc) Storage, Modeling, and Applications. Nanoscale Res. Lett..

[42] Zhou S., Xing Y., Xu Q., Yan Q., Liu P., Wei L., Niu W., Li F., You L., Pu Y. (2023). Planar Memristor and Artificial Synaptic Simulating Based on Two-Dimensional Layered Tungsten Oxychloride WO_2_Cl_2_. Appl. Phys. Lett..

[43] Li Y., Zhong Y., Zhang J., Xu L., Wang Q., Sun H., Tong H., Cheng X., Miao X. (2014). Activity-Dependent Synaptic Plasticity of a Chalcogenide Electronic Synapse for Neuromorphic Systems. Sci. Rep..

[44] Zhao M., Gao B., Tang J., Qian H., Wu H. (2020). Reliability of Analog Resistive Switching Memory for Neuromorphic Computing. Appl. Phys. Rev..

[45] Tsuruoka T., Valov I., Tappertzhofen S., van den Hurk J., Hasegawa T., Waser R., Aono M. (2015). Redox Reactions at Cu, Ag/Ta_2_O_5_ Interfaces and the Effects of Ta_2_O_5_ Film Density on the Forming Process in Atomic Switch Structures. Adv. Funct. Mater..

[46] Yalishev V.S., Yuldashev S.U., Kim Y.S., Park B.H. (2012). The Role of Zinc Vacancies in Bipolar Resistance Switching of Ag/ZnO/Pt Memory Structures. Nanotechnology.

[47] Chung Y.L., Cheng W.H., Jeng J.S., Chen W.C., Jhan S.A., Chen J.S. (2014). Joint Contributions of Ag Ions and Oxygen Vacancies to Conducting Filament Evolution of Ag/TaO_x_/Pt Memory Device. J. Appl. Phys..

[48] Tada M., Sakamoto T., Banno N., Okamoto K., Miyamura M., Iguchi N., Hada H. (2012). Improved Reliability and Switching Performance of Atom Switch by Using Ternary Cu-Alloy and RuTa Electrodes. Proceedings of the 2012 International Electron Devices Meeting.

[49] Liu T., Verma M., Kang Y., Orlowski M. (2012). Volatile Resistive Switching in Cu/TaOx/δ-Cu/Pt Devices. Appl. Phys. Lett..

[50] Chandrasekaran S., Simanjuntak F.M., Tsai T.-L., Lin C.-A., Tseng T.-Y. (2017). Effect of Barrier Layer on Switching Polarity of ZrO_2_-Based Conducting-Bridge Random Access Memory. Appl. Phys. Lett..

[51] Wu X., Mei S., Bosman M., Raghavan N., Zhang X., Cha D., Li K., Pey K.L. (2015). Evolution of Filament Formation in Ni/HfO_2_/SiO_x_/Si-Based RRAM Devices. Adv. Electron. Mater..

[52] Belmonte A., Witters T., Covello A., Vereecke G., Franquet A., Spampinato V., Kundu S., Mao M., Hody H., Kar G.S. (2019). Co Active Electrode Enhances CBRAM Performance and Scaling Potential. Proceedings of the 2019 IEEE International Electron Devices Meeting (IEDM).

[53] Qian K., Han X., Li H., Chen T., Lee P.S. (2020). Uncovering the Indium Filament Revolution in Transparent Bipolar ITO/SiO_x_/ITO Resistive Switching Memories. ACS Appl. Mater. Interfaces.

[54] Sun B., Han X., Xu R., Qian K. (2020). Uncovering the Indium Filament Formation and Dissolution in Transparent ITO/SiN_x_/ITO Resistive Random Access Memory. ACS Appl. Electron. Mater..

[55] Qian K., Tay R.Y., Lin M.-F., Chen J., Li H., Lin J., Wang J., Cai G., Nguyen V.C., Teo E.H.T. (2017). Direct Observation of Indium Conductive Filaments in Transparent, Flexible, and Transferable Resistive Switching Memory. ACS Nano.

[56] Rajasekaran S., Simanjuntak F.M., Panda D., Chandrasekaran S., Aluguri R., Saleem A., Tseng T.-Y. (2020). Fast, Highly Flexible, and Transparent TaO_x_ -Based Environmentally Robust Memristors for Wearable and Aerospace Applications. ACS Appl. Electron. Mater..

[57] Shi Y., Nguyen L., Oh S., Liu X., Koushan F., Jameson J.R., Kuzum D. (2018). Neuroinspired Unsupervised Learning and Pruning with Subquantum CBRAM Arrays. Nat. Commun..

[58] Wang H., Yan X., Wang S., Lu N. (2021). High-Stability Memristive Devices Based on Pd Conductive Filaments and Its Applications in Neuromorphic Computing. ACS Appl. Mater. Interfaces.

[59] Cooper D., Baeumer C., Bernier N., Marchewka A., La Torre C., Dunin-Borkowski R.E., Menzel S., Waser R., Dittmann R. (2017). Anomalous Resistance Hysteresis in Oxide ReRAM: Oxygen Evolution and Reincorporation Revealed by In Situ TEM. Adv. Mater..

[60] Kim M.J., Jeon D.S., Park J.H., Kim T.G. (2015). Bipolar Resistive Switching Characteristics in Tantalum Nitride-Based Resistive Random Access Memory Devices. Appl. Phys. Lett..

[61] Kim H.-D., Yun M.J., Kim T.G. (2015). Forming-Free Resistive Switching Characteristics and Improved Reliability in Sub-Stoichiometric NbN*_x_* Films. Phys. Status Solidi (RRL)—Rapid Res. Lett..

[62] Chang Y.-C., Wang Y.-H. (2014). Resistive Switching Behavior in Gelatin Thin Films for Nonvolatile Memory Application. ACS Appl. Mater. Inter..

[63] Kang Y., Liu T., Potnis T., Orlowski M.K. (2013). Composite Cu/VO and VO/Cu Nanofilaments in Cu/Ta_2_O_5_/Pt Devices. ECS Solid State Lett..

[64] Chang C.F., Chen J.Y., Huang C.W., Chiu C.H., Lin T.Y., Yeh P.H., Wu W.W. (2017). Direct Observation of Dual-Filament Switching Behaviors in Ta_2_O_5_-Based Memristors. Small.

[65] Sun H., Liu Q., Li C., Long S., Lv H., Bi C., Huo Z., Li L., Liu M. (2014). Direct Observation of Conversion Between Threshold Switching and Memory Switching Induced by Conductive Filament Morphology. Adv. Funct. Mater..

[66] Peng C.-N., Wang C.-W., Chan T.-C., Chang W.-Y., Wang Y.-C., Tsai H.-W., Wu W.-W., Chen L.-J., Chueh Y.-L. (2012). Resistive Switching of Au/ZnO/Au Resistive Memory: An in Situ Observation of Conductive Bridge Formation. Nanoscale Res. Lett..

[67] Tian X., Wang L., Wei J., Yang S., Wang W., Xu Z., Bai X. (2014). Filament Growth Dynamics in Solid Electrolyte-Based Resistive Memories Revealed by in Situ TEM. Nano Res..

[68] Yang Y., Gao P., Li L., Pan X., Tappertzhofen S., Choi S., Waser R., Valov I., Lu W.D. (2014). Electrochemical Dynamics of Nanoscale Metallic Inclusions in Dielectrics. Nat. Commun..

[69] Waser R., Dittmann R., Staikov G., Szot K. (2009). Redox-Based Resistive Switching Memories—Nanoionic Mechanisms, Prospects, and Challenges. Adv. Mater..

[70] Zhang Z., Gao B., Fang Z., Wang X., Tang Y., Sohn J., Wong H.-S.P., Wong S.S., Lo G.-Q. (2015). All-Metal-Nitride RRAM Devices. IEEE Electron. Device Lett..

[71] Chai Y., Wu Y., Takei K., Chen H.-Y., Yu S., Chan P.C.H., Javey A., Wong H.-S.P. (2010). Resistive Switching of Carbon-Based RRAM with CNT Electrodes for Ultra-Dense Memory. Proceedings of the 2010 International Electron Devices Meeting.

[72] Russo P., Xiao M., Zhou N.Y. (2019). Electrochemical Oxidation Induced Multi-Level Memory in Carbon-Based Resistive Switching Devices. Sci. Rep..

[73] Zhang Y., Mao G.-Q., Zhao X., Li Y., Zhang M., Wu Z., Wu W., Sun H., Guo Y., Wang L. (2021). Evolution of the Conductive Filament System in HfO2-Based Memristors Observed by Direct Atomic-Scale Imaging. Nat. Commun..

[74] Yao J., Zhong L., Natelson D., Tour J.M. (2012). In Situ Imaging of the Conducting Filament in a Silicon Oxide Resistive Switch. Sci. Rep..

[75] Liu T., Verma M., Kang Y., Orlowski M.K. (2012). Coexistence of Bipolar and Unipolar Switching of Cu and Oxygen Vacancy Nanofilaments in Cu/TaO_x_/Pt Resistive Devices. ECS Solid. State Lett..

[76] Huang C.-H., Huang J.-S., Lai C.-C., Huang H.-W., Lin S.-J., Chueh Y.-L. (2013). Manipulated Transformation of Filamentary and Homogeneous Resistive Switching on ZnO Thin Film Memristor with Controllable Multistate. ACS Appl. Mater. Interfaces.

[77] Tambunan O.T., Parwanta K.J., Acharya S.K., Lee B.W., Jung C.U., Kim Y.S., Park B.H., Jeong H., Park J.Y., Cho M.R. (2014). Resistance Switching in Epitaxial SrCoO_x_ Thin Films. Appl. Phys. Lett..

[78] Yao L., Inkinen S., van Dijken S. (2017). Direct Observation of Oxygen Vacancy-Driven Structural and Resistive Phase Transitions in La_2/3_Sr_1/3_MnO_3_. Nat. Commun..

[79] Kim H.G., Nallagatla V.R., Kwon D.H., Jung C.U., Kim M. (2020). In Situ Observations of Topotactic Phase Transitions in a Ferrite Memristor. J. Appl. Phys..

[80] Wong H.-S.P., Raoux S., Kim S., Liang J., Reifenberg J.P., Rajendran B., Asheghi M., Goodson K.E. (2010). Phase Change Memory. Proc. IEEE.

[81] Wang L., Liao W., Wong S.L., Yu Z.G., Li S., Lim Y., Feng X., Tan W.C., Huang X., Chen L. (2019). Artificial Synapses Based on Multiterminal Memtransistors for Neuromorphic Application. Adv. Funct. Mater..

[82] Sangwan V.K., Lee H.-S., Bergeron H., Balla I., Beck M.E., Chen K.-S., Hersam M.C. (2018). Multi-Terminal Memtransistors from Polycrystalline Monolayer Molybdenum Disulfide. Nature.

[83] Yan X., Qian J.H., Sangwan V.K., Hersam M.C. (2022). Progress and Challenges for Memtransistors in Neuromorphic Circuits and Systems. Adv. Mater..

[84] Berdan R., Vasilaki E., Khiat A., Indiveri G., Serb A., Prodromakis T. (2016). Emulating Short-Term Synaptic Dynamics with Memristive Devices. Sci. Rep..

[85] Löwel S., Singer W. (1992). Selection of Intrinsic Horizontal Connections in the Visual Cortex by Correlated Neuronal Activity. Science.

[86] Ringo J.L. (1991). Neuronal Interconnection as a Function of Brain Size. Brain Behav. Evol..

[87] Neveu D., Zucker R.S. (1996). Long-Lasting Potentiation and Depression without Presynaptic Activity. J. Neurophysiol..

[88] Bliss T.V.P., Cooke S.F. (2011). Long-Term Potentiation and Long-Term Depression: A Clinical Perspective. Clinics.

[89] Ohno T., Hasegawa T., Tsuruoka T., Terabe K., Gimzewski J.K., Aono M. (2011). Short-Term Plasticity and Long-Term Potentiation Mimicked in Single Inorganic Synapses. Nat. Mater..

[90] Wu C.-H., Lin S.-K., Pan C.-H., Chen P.-H., Lin W.-Y., Chang T.-C., Tsai T.-M., Xu Y.-L., Shih C.-C., Lin Y.-S. (2018). Analyzing Electric Field Effect by Applying an Ultra-Short Time Pulse Condition in Hafnium Oxide-Based RRAM. IEEE Electron. Device Lett..

[91] Hennig M.H. (2013). Theoretical Models of Synaptic Short Term Plasticity. Front. Comput. Neurosci..

[92] Kurenkov A., DuttaGupta S., Zhang C., Fukami S., Horio Y., Ohno H. (2019). Artificial Neuron and Synapse Realized in an Antiferromagnet/Ferromagnet Heterostructure Using Dynamics of Spin–Orbit Torque Switching. Adv. Mater..

[93] Simanjuntak F.M., Hsu C.-L., Abbey T., Chang L.-Y., Rajasekaran S., Prodromakis T., Tseng T.-Y. (2021). Conduction Channel Configuration Controlled Digital and Analog Response in TiO_2_-Based Inorganic Memristive Artificial Synapses. APL Mater..

[94] Simanjuntak F.M., Ohno T., Chandrasekaran S., Tseng T.-Y., Samukawa S. (2020). Neutral Oxygen Irradiation Enhanced Forming-Less ZnO-Based Transparent Analog Memristor Devices for Neuromorphic Computing Applications. Nanotechnology.

[95] Juliano H., Limantoro S.E., Simanjuntak F.M., Tseng T.-Y. (2025). Metal Interdiffusion Enhanced WO_x_/CuO_x_ Heterojunction Optoelectronic Memristive Synapses for Face Recognition Application. APL Mater..

[96] Simanjuntak F.M., Chandrasekaran S., Lin C.-C., Tseng T.-Y. (2019). ZnO_2_/ZnO Bilayer Switching Film for Making Fully Transparent Analog Memristor Devices. APL Mater..

[97] Chen P.-Y., Lin B., Wang I.-T., Hou T.-H., Ye J., Vrudhula S., Seo J., Cao Y., Yu S. (2015). Mitigating Effects of Non-Ideal Synaptic Device Characteristics for on-Chip Learning. Proceedings of the 2015 IEEE/ACM International Conference on Computer-Aided Design (ICCAD).

[98] Lastras-Montaño M.A., Cheng K.-T. (2018). Resistive Random-Access Memory Based on Ratioed Memristors. Nat. Electron..

[99] Gao B., Bi Y., Chen H.-Y., Liu R., Huang P., Chen B., Liu L., Liu X., Yu S., Wong H.-S.P. (2014). Ultra-Low-Energy Three-Dimensional Oxide-Based Electronic Synapses for Implementation of Robust High-Accuracy Neuromorphic Computation Systems. ACS Nano.

[100] Ahmed T., Tahir M., Low M.X., Ren Y., Tawfik S.A., Mayes E.L.H., Kuriakose S., Nawaz S., Spencer M.J.S., Chen H. (2021). Fully Light-Controlled Memory and Neuromorphic Computation in Layered Black Phosphorus. Adv. Mater..

[101] Wang Y., Lv Z., Chen J., Wang Z., Zhou Y., Zhou L., Chen X., Han S.-T. (2018). Photonic Synapses Based on Inorganic Perovskite Quantum Dots for Neuromorphic Computing. Adv. Mater..

[102] Tran M.D., Kim H., Kim J.S., Doan M.H., Chau T.K., Vu Q.A., Kim J.-H., Lee Y.H. (2019). Two-Terminal Multibit Optical Memory via van Der Waals Heterostructure. Adv. Mater..

[103] Pereira M.E., Martins R., Fortunato E., Barquinha P., Kiazadeh A. (2023). Recent Progress in Optoelectronic Memristors for Neuromorphic and In-Memory Computation. Neuromorphic Comput. Eng..

[104] Vashishtha P., Kofler C., Verma A.K., Giridhar S.P., Tollerud J.O., Dissanayake N.S.L., Gupta T., Sehrawat M., Aggarwal V., Mayes E.L.H. (2025). Epitaxial Interface-Driven Photoresponse Enhancement in Monolayer WS_2_ –MoS_2_ Lateral Heterostructures. Adv. Funct. Mater..

[105] Prajapat P., Vashishtha P., Gupta G. (2025). High-Temperature Resilient Neuromorphic Device Based on Optically Configured Monolayer MoS_2_ for Cognitive Computing. Small.

[106] Zhang X., Zhao X., Shan X., Tian Q., Wang Z., Lin Y., Xu H., Liu Y. (2021). Humidity Effect on Resistive Switching Characteristics of the CH_3_NH_3_PbI_3_ Memristor. ACS Appl. Mater. Interfaces.

[107] Haghshenas Gorgabi F., Morant-Miñana M.C., Zafarkish H., Abbaszadeh D., Asadi K. (2023). Graphene Memristors Based on Humidity-Mediated Reduction of Graphene Oxide. J. Mater. Chem. C Mater..

[108] Xia Z., Sun X., Wang Z., Meng J., Jin B., Wang T. (2025). Low-Power Memristor for Neuromorphic Computing: From Materials to Applications. Nanomicro Lett..

[109] Wu Y., Chang A., Chen W., Ercan E., Weng Y., Lin B., Liu C., Lin Y., Chen W. (2024). High-Performance Synaptic Phototransistor Using A Photoactive Self-Assembled Layer toward Ultralow Energy Consumption. Adv. Opt. Mater..

[110] Shrivastava S., Juliano H., Uong P.A.L., Tseng T.-Y. (2025). Recent Developments on Neuromorphic Optoelectronic Memristors Based on Metal Oxide Semiconductors: A Review. APL Electron. Devices.

[111] Islam M.M., Dev D., Krishnaprasad A., Tetard L., Roy T. (2020). Optoelectronic Synapse Using Monolayer MoS_2_ Field Effect Transistors. Sci. Rep..

[112] Shrivastava S., Limantoro S.E., Juliano H., Tseng T.-Y. (2025). Perovskite Oxide Based Transparent Neuromorphic Photoelectric Memristor for Artificial Ocular System. APL Mater..

[113] Xie P., Li D., Yip S., Ho J.C. (2024). Emerging Optoelectronic Artificial Synapses and Memristors Based on Low-Dimensional Nanomaterials. Appl. Phys. Rev..

[114] Dun G., Li Y., Zhang H., Wu F., Tan X., Qin K., He Y., Wang Z., Wang Y., Lu T. (2025). All-in-one Perovskite Memristor with Tunable Photoresponsivity. InfoMat.

[115] Tang B., Veluri H., Li Y., Yu Z.G., Waqar M., Leong J.F., Sivan M., Zamburg E., Zhang Y.-W., Wang J. (2022). Wafer-Scale Solution-Processed 2D Material Analog Resistive Memory Array for Memory-Based Computing. Nat. Commun..

[116] Khan R., Rehman N.U., Kalluri S., Elumalai S., Saritha A., Fakhar-e-alam M., Ikram M., Abdullaev S., Rahman N., Sangaraju S. (2025). 2D MoTe_2_ Memristors for Energy-Efficient Artificial Synapses and Neuromorphic Applications. Nanoscale.

[117] Zhang W., Gao H., Deng C., Lv T., Hu S., Wu H., Xue S., Tao Y., Deng L., Xiong W. (2021). An Ultrathin Memristor Based on a Two-Dimensional WS_2_/MoS_2_ Heterojunction. Nanoscale.

[118] Khot A.C., Dongale T.D., Nirmal K.A., Sung J.H., Lee H.J., Nikam R.D., Kim T.G. (2022). Amorphous Boron Nitride Memristive Device for High-Density Memory and Neuromorphic Computing Applications. ACS Appl. Mater. Interfaces.

[119] Roldan J.B., Maldonado D., Aguilera-Pedregosa C., Moreno E., Aguirre F., Romero-Zaliz R., García-Vico A.M., Shen Y., Lanza M. (2022). Spiking Neural Networks Based on Two-Dimensional Materials. NPJ 2D Mater. Appl..

[120] Wang K., Chen J., Yan X. (2021). MXene Ti_3_C_2_ Memristor for Neuromorphic Behavior and Decimal Arithmetic Operation Applications. Nano Energy.

[121] Caporale N., Dan Y. (2008). Spike Timing–Dependent Plasticity: A Hebbian Learning Rule. Annu. Rev. Neurosci..

[122] Hebb D.O. (1949). The Organization of Behavior; A Neuropsychological Theory.

[123] Saïghi S., Mayr C.G., Serrano-Gotarredona T., Schmidt H., Lecerf G., Tomas J., Grollier J., Boyn S., Vincent A.F., Querlioz D. (2015). Plasticity in Memristive Devices for Spiking Neural Networks. Front. Neurosci..

[124] Bi G., Poo M. (2001). Synaptic Modification by Correlated Activity: Hebb’s Postulate Revisited. Annu. Rev. Neurosci..

[125] Huang P., Li Z., Dong Z., Han R., Zhou Z., Zhu D., Liu L., Liu X., Kang J. (2019). Binary Resistive-Switching-Device-Based Electronic Synapse with Spike-Rate-Dependent Plasticity for Online Learning. ACS Appl. Electron. Mater..

[126] Li Y., Zhong Y., Xu L., Zhang J., Xu X., Sun H., Miao X. (2013). Ultrafast Synaptic Events in a Chalcogenide Memristor. Sci. Rep..

[127] Buchanan K.A. (2010). The Activity Requirements for Spike Timing-Dependent Plasticity in the Hippocampus. Front. Synaptic Neurosci..

[128] Li G., Xie D., Zhong H., Zhang Z., Fu X., Zhou Q., Li Q., Ni H., Wang J., Guo E. (2022). Photo-Induced Non-Volatile VO_2_ Phase Transition for Neuromorphic Ultraviolet Sensors. Nat. Commun..

[129] Javanshir A., Nguyen T.T., Mahmud M.A.P., Kouzani A.Z. (2022). Advancements in Algorithms and Neuromorphic Hardware for Spiking Neural Networks. Neural Comput..

[130] Iyer L.R., Chua Y., Li H. (2021). Is Neuromorphic MNIST Neuromorphic? Analyzing the Discriminative Power of Neuromorphic Datasets in the Time Domain. Front. Neurosci..

[131] Siddique A., Vai M.I., Pun S.H. (2023). A Low Cost Neuromorphic Learning Engine Based on a High Performance Supervised SNN Learning Algorithm. Sci. Rep..

[132] Bayat F.M., Prezioso M., Chakrabarti B., Nili H., Kataeva I., Strukov D. (2018). Implementation of Multilayer Perceptron Network with Highly Uniform Passive Memristive Crossbar Circuits. Nat. Commun..

[133] Jiang H. (2020). Artificial Eye Boosted by Hemispherical Retina. Nature.

[134] Merabet L.B. (2011). Building the Bionic Eye: An Emerging Reality and Opportunity. Progress in Brain Research.

[135] Berco D., Shenp Ang D. (2019). Recent Progress in Synaptic Devices Paving the Way toward an Artificial Cogni-Retina for Bionic and Machine Vision. Adv. Intell. Syst..

[136] Seo S., Jo S., Kim S., Shim J., Oh S., Kim J., Heo K., Choi J.-W., Choi C., Oh S. (2018). Artificial Optic-Neural Synapse for Colored and Color-Mixed Pattern Recognition. Nat. Commun..

[137] Lee Y., Rhee H., Kim G., Cheong W.H., Kim D.H., Song H., Kay S.N., Lee J., Kim K.M. (2025). Flexible Self-Rectifying Synapse Array for Energy-Efficient Edge Multiplication in Electrocardiogram Diagnosis. Nat. Commun..

[138] Chen S., Zhang T., Tappertzhofen S., Yang Y., Valov I. (2023). Electrochemical-Memristor-Based Artificial Neurons and Synapses—Fundamentals, Applications, and Challenges. Adv. Mater..

[139] Lan J., Chen Y., Cao Z., Wang K., Lu Q., Ren F., Lv Y., Sun B., Wu R. (2025). Memristor-Based Intelligent Systems for Sensing, Computing, and Therapeutic Integration Applications. Mater. Today Adv..

[140] Dias C., Castro D., Aroso M., Ventura J., Aguiar P. (2022). Memristor-Based Neuromodulation Device for Real-Time Monitoring and Adaptive Control of Neuronal Populations. ACS Appl. Electron. Mater..

[141] Cao Z., Xiang L., Sun B., Gao K., Yu J., Zhou G., Duan X., Yan W., Lin F., Li Z. (2024). A Reversible Implantable Memristor for Health Monitoring Applications. Mater. Today Bio.

[142] Mikhaylov A., Pimashkin A., Pigareva Y., Gerasimova S., Gryaznov E., Shchanikov S., Zuev A., Talanov M., Lavrov I., Demin V. (2020). Neurohybrid Memristive CMOS-Integrated Systems for Biosensors and Neuroprosthetics. Front. Neurosci..

[143] Prieto-Avalos G., Cruz-Ramos N.A., Alor-Hernández G., Sánchez-Cervantes J.L., Rodríguez-Mazahua L., Guarneros-Nolasco L.R. (2022). Wearable Devices for Physical Monitoring of Heart: A Review. Biosensors.

[144] Li H., Kumar D., El-Atab N. (2025). A Memristor-Based Event-Driven Reservoir Computing System for Cardiac Arrhythmia Detection. Adv. Intell. Syst..

[145] Wang Z., Yang D.W., Liu Z., Yan E., Sun H., Ge N., Hu M., Wu W. (2025). Multi-Diseases Detection with Memristive System on Chip. Adv. Intell. Syst..

[146] Ni K., Grisafe B., Chakraborty W., Saha A.K., Dutta S., Jerry M., Smith J.A., Gupta S., Datta S. (2018). In-Memory Computing Primitive for Sensor Data Fusion in 28 Nm HKMG FeFET Technology. Proceedings of the 2018 IEEE International Electron Devices Meeting (IEDM).

